# Supplementary material for: The Fast and the Furriest: Investigating the Rate of Selection on Mammalian Toxins
Source: Toxins (Basel). 2022 Dec 1;14(12):842. doi: 10.3390/toxins14120842 (PMC9782207; doi:10.3390/toxins14120842)
Supplement: Supplementary file 1 [file toxins-14-00842-s001.zip › TheFastandTheFurriest_Supplemntary2022.pdf]

The Fast and the Furriest: Investigating the Rate of Selection on Mammalian Toxins

Leah Lucy Joscelyne Fitzpatrick, Vincent Nijman, Rodrigo Ligabue-Braun, K. Anne-Isola Nekaris

The following supplementary data includes details on the number of sequences used, full FEL, FUBAR and MEME results, all reported PAML results and significance of calculated LRTs, TreeSAAP results and model selection.

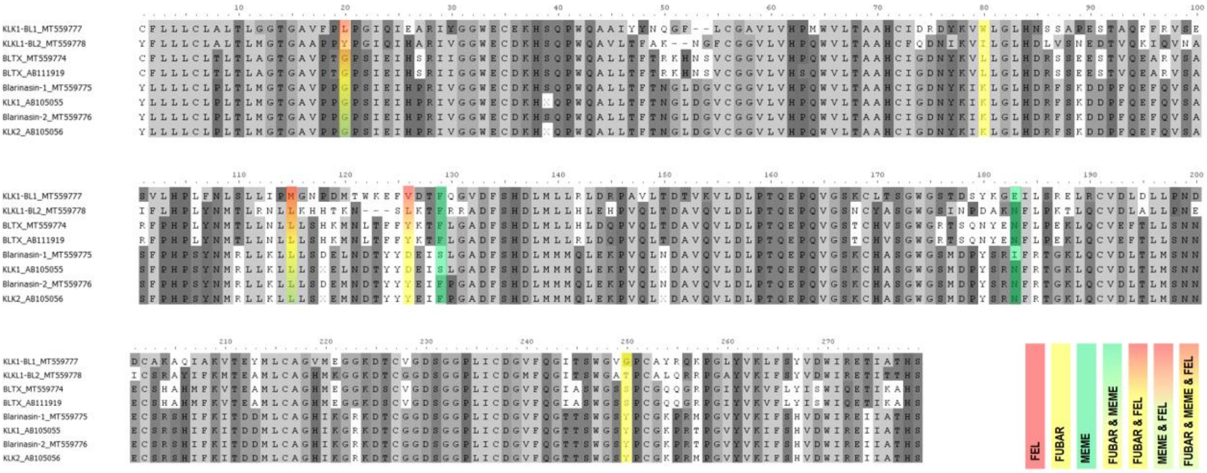

Figure S1. Amino acid alignment of KLK1 *Blarina*, with diversifying selection highlighted according to each programme. Red represents FEL, Yellow represents FUBAR and Green represents MEME. Overlapping sites with partial or complete programme agreement are colored as mixed hues.

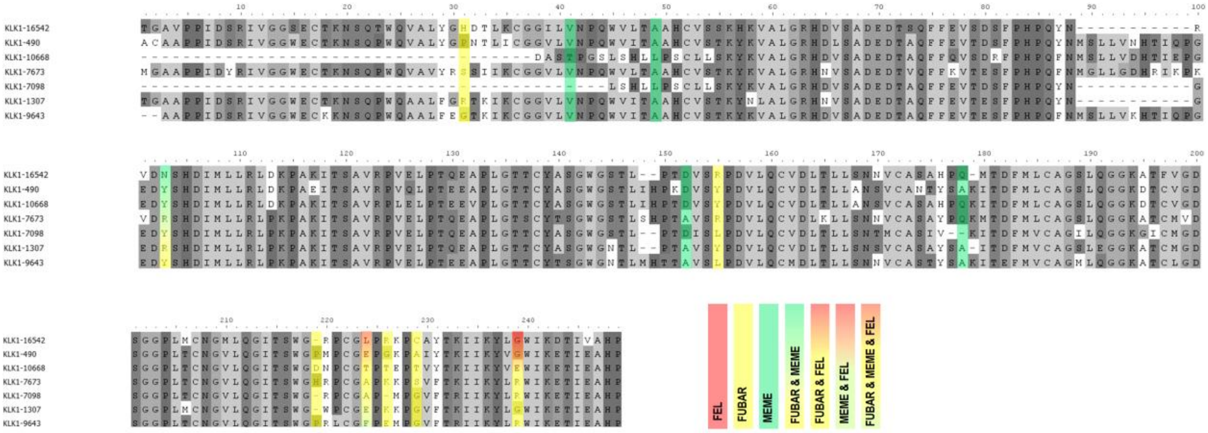

Figure S2. Amino acid alignment of KLK1 *Solenodon*, with diversifying selection highlighted according to each programme. Red represents FEL, Yellow represents FUBAR and Green represents MEME. Overlapping sites with partial or complete programme agreement are colored as mixed hues.

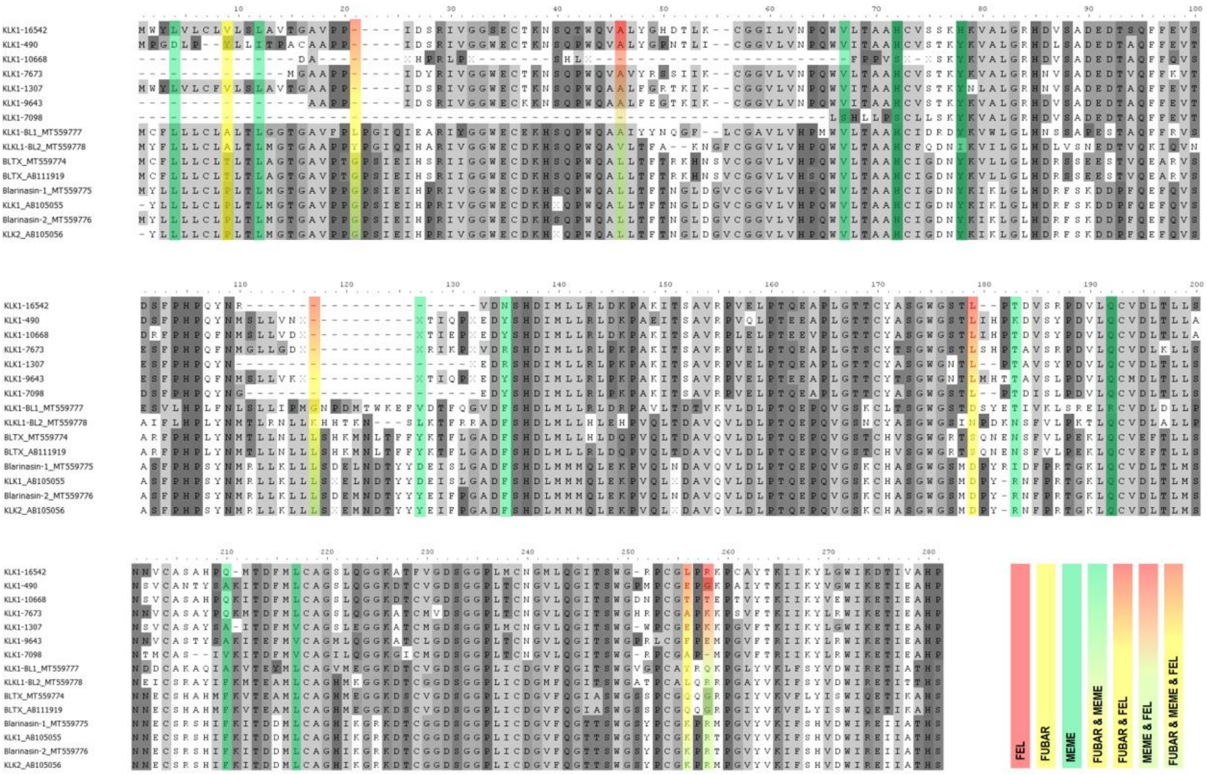

Figure S3. Amino acid alignment of KLK1 Eulipotyphla, with diversifying selection highlighted according to each programme. Red represents FEL, Yellow represents FUBAR and Green represents MEME. Overlapping sites with partial or complete programme agreement are colored as mixed hues.

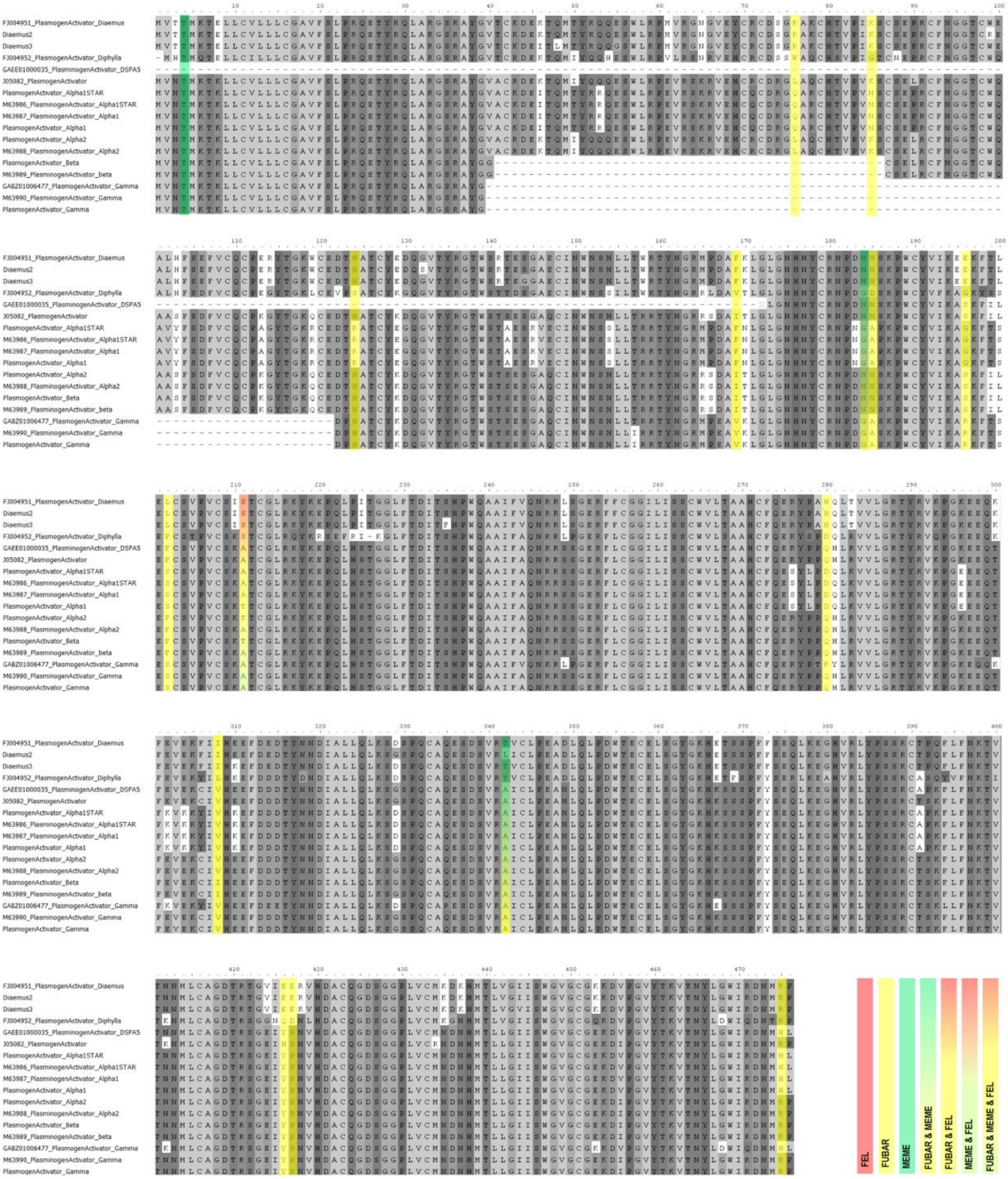

Figure S4. Amino acid alignment of Plasminogen Activator, with diversifying selection highlighted according to each programme. Red represents FEL, Yellow represents FUBAR and Green represents MEME. Overlapping sites with partial or complete programme agreement are colored as mixed hues.

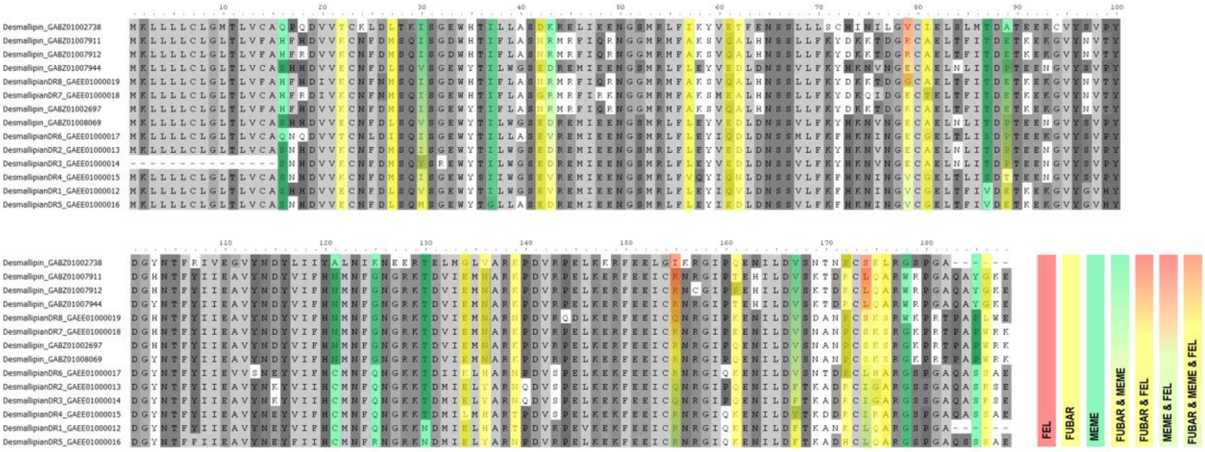

Figure S5. Amino acid alignment of Desmallipins, with diversifying selection highlighted according to each programme. Red represents FEL, Yellow represents FUBAR and Green represents MEME. Overlapping sites with partial or complete programme agreement are colored as mixed hues.

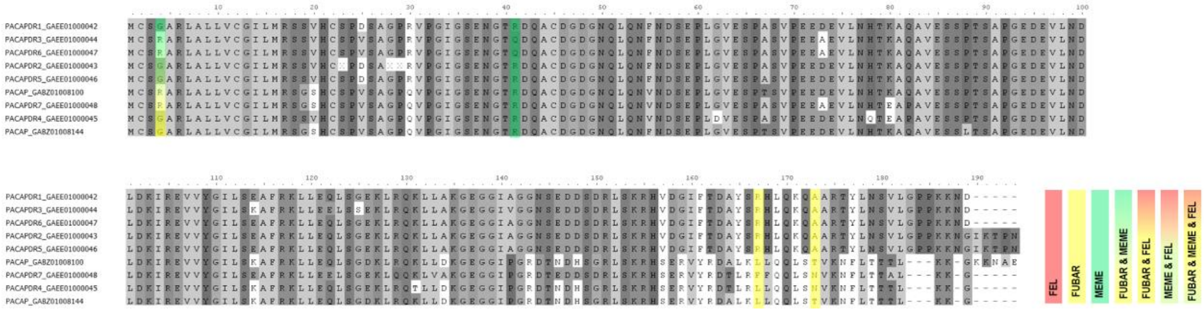

Figure S6. Amino acid alignment of PACAP, with diversifying selection highlighted according to each programme. Red represents FEL, Yellow represents FUBAR and Green represents MEME. Overlapping sites with partial or complete programme agreement are colored as mixed hues.

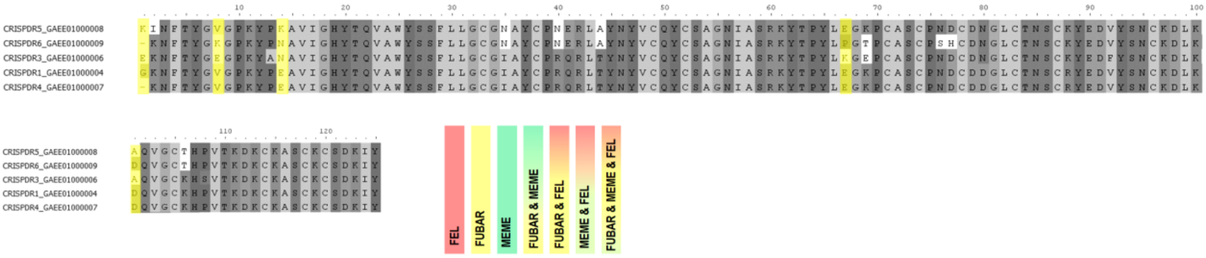

Figure S7. Amino acid alignment of CRiSP *Desmodus*, with diversifying selection highlighted according to each programme. Red represents FEL, Yellow represents FUBAR and Green represents MEME. Overlapping sites with partial or complete programme agreement are colored as mixed hues.

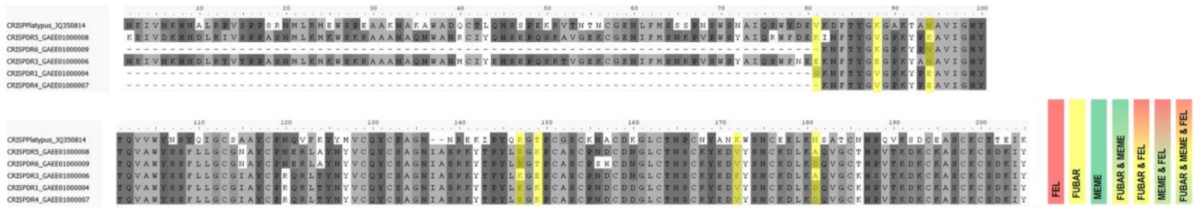

Figure S8. Amino acid alignment of CRiSP *Desmodus* and *Ornithorhynchus*, with diversifying selection highlighted according to each programme. Red represents FEL, Yellow represents FUBAR and Green represents MEME. Overlapping sites with partial or complete programme agreement are colored as mixed hues.

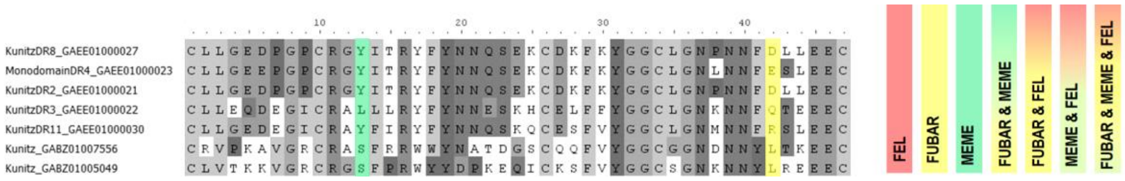

Figure S9. Amino acid alignment of Kunitz Domain One, with diversifying selection highlighted according to each programme. Red represents FEL, Yellow represents FUBAR and Green represents MEME. Overlapping sites with partial or complete programme agreement are colored as mixed hues.

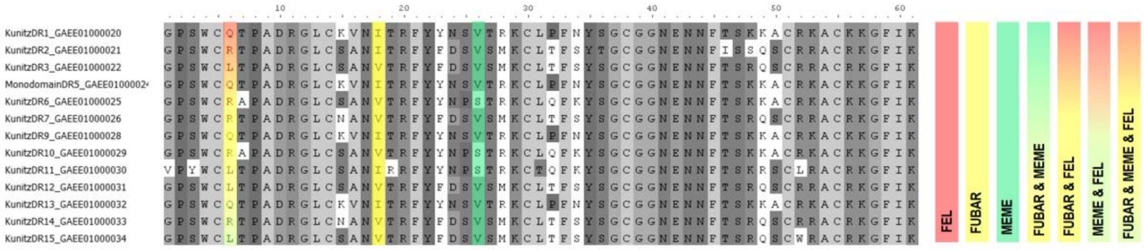

Figure S10. Amino acid alignment of Kunitz Domain Two, with diversifying selection highlighted according to each programme. Red represents FEL, Yellow represents FUBAR and Green represents MEME. Overlapping sites with partial or complete programme agreement are colored as mixed hues.

Table S1: The number of sequences and length of each alignment. Length of alignment does not include STOP codons, which were removed prior to analysis. Each alignment is included within Appendix 1.

Toxin name	Number of sequences	Length of alignment after TrimAI	Species present
Kallikren-1(KLK1) *Blarina*	8	837	Short tailed shrew (*Blarina brevicauda*)
Kallikren-1(KLK1) *Solenodon*	7	747	Hispaniolan solenodon (*Solenodon paradoxus*)
Kallikrein-1 (KLK1) Eulipotyphla	15	843	Short tailed shrew (*Blarina brevicauda*)<br><br>Hispaniolan solenodon (*Solenodon paradoxus*)
Kallikren-1 (KLK1) All	16	789	Short tailed shrew (*Blarina brevicauda*)<br><br>Hispaniolan solenodon (*Solenodon paradoxus*)<br><br>Common Vampire Bat (*Desmodus rotundus*)
Plasminogen Activator	17	1428	Hairy legged vampire bat (*Diphylla ecaudata*)<br><br>White winged vampire bat (*Diaemus youngi*)<br><br>Common vampire bat (*Desmodus rotundus*)
Desmallipins (*Desmodus* allergen-related lipocalins)	14	564	Common Vampire Bat (*Desmodus rotundus*)
PACAP (Pituitary Adenylate Cyclase-Activating Polypeptide)	9	582	Common Vampire Bat (*Desmodus rotundus*)
Cysteine-rich secretory proteins (CRiSP) *Desmodus*	5	375	Common Vampire Bat (*Desmodus rotundus*)
Cysteine-rich secretory proteins (CRiSP)<br>*Desmodus* and *Ornithorhynchus*	6	615	Common Vampire Bat (*Desmodus rotundus*)<br><br>Platypus (*Ornithorhynchus anatinus*)
Kunitz Domain One	7	141	Common Vampire Bat (*Desmodus rotundus*)
Kunitz Domain Two	13	183	Common Vampire Bat (*Desmodus rotundus*)

Table S2: FEL results for each alignment including number of sites identified to be under significant diversifying or purifying selection and at which site in each alignment.					
Toxin	Species	Sites identified under diversifying selection	Sites number	Sites identified under purifying selection	Sites number
Kallikren-1(KLK1) *Blarina*	Short tailed shrew (*Blarina brevicauda*)	3	20, 116, 126	26	13, 14, 23, 29, 38, 39, 83, 94, 104, 136, 146, 150, 151, 160, 173, 175, 191, 199, 202, 209, 211, 216, 224, 233, 238, 267
Kallikren-1(KLK1) *Solenodon*	Hispaniolan solenodon (*Solenodon paradoxus*)	2	224, 239	19	16, 21, 52, 53, 75, 76, 86, 104, 118, 139, 144, 154, 166, 186, 192, 196, 204, 223, 228
Kallikrein-1 (KLK1) Eulipotyphla	Short tailed shrew (*Blarina brevicauda*) Hispaniolan solenodon (*Solenodon paradoxus*)	6	21, 46, 117, 179, 256, 258	47	2,6,15,17,24,26,29,32,35,38,39,40,61,66,73,85,95,105,107,134,136,137,147,151,153,161,171,174,176,193,198,201,204,211,213,218,224,226,228,235,236,239,241,244,260,277,280
Kallikren-1 (KLK1) All	Short tailed shrew (*Blarina brevicauda*) Hispaniolan solenodon (*Solenodon paradoxus*) Common Vampire Bat (*Desmodus rotundus*)	4	83, 179, 238, 240	48	2, 6, 17, 21, 25, 27, 30, 33, 34, 35, 50, 53, 54, 59, 66, 78, 88, 98, 100, 116, 118, 119, 129, 133, 135, 143, 153, 156, 158, 175, 180, 183, 186, 193, 195, 200, 206, 208, 210, 218, 221, 223, 226, 232, 235, 242, 259, 262

Plasminogen Activator	Hairy legged vampire bat (*Diphylla ecaudata*)<br><br>White winged vampire bat (*Diaemus youngi*)<br><br>Common vampire bat (*Desmodus rotundus*)	1	211	22	44, 73, 79, 83, 128, 135, 153, 154, 179, 257, 258, 259, 307, 311, 344, 363, 381, 392, 420, 455, 465, 473
Desmallipins (*Desmodus* allergen-related lipocalins)	Common Vampire Bat (*Desmodus rotundus*)	3	79, 155, 174	13	19, 41, 64,65, 73, 83, 88, 105, 118, 151, 158, 173, 180
PACAP (Pituitary Adenylate Cyclase-Activating Polypeptide)	Common Vampire Bat (*Desmodus rotundus*)	0	N/A	10	66, 81, 92, 99, 144, 163, 186, 187, 189, 193
Cysteine-rich secretory proteins (CRiSP) *Desmodus*	Common Vampire Bat (*Desmodus rotundus*)	0	N/A	3	6, 11, 42
Cysteine-rich secretory proteins (CRiSP) *Desmodus* and *Ornithorhynchus*	Common Vampire Bat (*Desmodus rotundus*)<br>Platypus (*Ornithorhynchus anatinus*)	0	N/A	19	2, 7, 60, 75, 86, 91, 99, 100, 117, 129, 131, 135, 153, 162, 163, 187, 194, 199, 200
Kunitz Domain One	Common Vampire Bat (*Desmodus rotundus*)	0	N/A	12	16, 19, 29, 31, 33, 34, 37, 39, 40, 45, 46, 47

Kunitz Domain Two	Common Vampire Bat (*Desmodus rotundus*)	1	6	2	8, 17

Table S3: FUBAR results for each alignment including number of sites identified to be under significant diversifying or purifying selection and at which site in each alignment.					
Toxin	Species	Sites identified under diversifying selection	Sites number	Sites identified under purifying selection	Sites number
Kallikren-1(KLK1) *Blarina*	Short tailed shrew (*Blarina brevicauda*)	5	20, 80, 116, 126, 250	23	13, 14, 19, 23, 38, 39, 83, 100, 104, 146, 150, 151, 152, 173, 199, 202, 209, 211, 216, 224, 233, 238, 267
Kallikren-1(KLK1) *Solenodon*	Hispaniolan solenodon (*Solenodon paradoxus*)	8	31, 103, 155, 219, 224, 226, 229, 239	16	16, 21, 53, 76, 86, 96, 104, 137, 154, 166, 186, 192, 196, 204, 223, 228
Kallikrein-1 (KLK1) Eulipotyphla	Short tailed shrew (*Blarina brevicauda*) Hispaniolan solenodon (*Solenodon paradoxus*)	5	9, 21, 117, 179, 256	35	6, 15, 17, 24, 32, 35, 39, 40, 61, 73, 85, 95, 105, 107, 134, 136, 137, 147, 151, 153, 171, 174, 176, 198, 201, 204, 211, 213, 218, 224, 226, 228, 236, 244, 260,
Kallikren-1 (KLK1) All	Short tailed shrew (*Blarina brevicauda*) Hispaniolan solenodon (*Solenodon paradoxus*) Common Vampire Bat (*Desmodus rotundus*)	3	169, 179, 238	47	2, 17, 20, 21, 25, 27, 30, 33, 34, 35, 53, 54, 59, 66, 67, 78, 88, 98, 100, 116, 118, 119, 129, 133, 135, 153, 156, 158, 163, 180, 183, 186, 193, 195, 200, 206, 208, 210, 218, 221, 223, 226, 232, 235, 242, 259, 262

Plasminogen Activator	Hairy legged vampire bat (*Diphylla ecaudata*)<br><br>White winged vampire bat (*Diaemus youngi*)<br><br>Common vampire bat (*Desmodus rotundus*)	15	76, 85, 124, 169, 184, 185, 196, 202, 211, 280, 308, 342, 416, 417, 475	8	154, 179, 257, 258, 311, 344, 368, 392
Desmallipins (Desmodus allergen-related lipocalins)	Common Vampire Bat (*Desmodus rotundus*)	22	22, 27, 30, 42, 43, 57, 61, 62, 79, 81, 89, 134, 136, 139, 155, 161, 167, 172, 174, 175, 176, 186	8	19, 64, 73, 88, 105, 118, 170, 184
PACAP (Pituitary Adenylate Cyclase-Activating Polypeptide)	Common Vampire Bat (*Desmodus rotundus*)	3	4, 167, 173	5	66, 81, 99, 144, 189
Cysteine-rich secretory proteins (CRiSP) *Desmodus*	Common Vampire Bat (*Desmodus rotundus*)	5	1, 8, 14, 67, 101	2	6, 11
Cysteine-rich secretory proteins (CRiSP) All	Common Vampire Bat (*Desmodus rotundus*)<br><br>Platypus (*Ornithorhynchus anatinus*)	7	81, 88, 94, 147, 149, 172, 181	2	91, 133

Kunitz Domain One	Common Vampire Bat (*Desmodus rotundus*)	1	42	7	16, 29, 31, 33, 37, 39, 40
Kunitz Domain Two (Chiroptera)	Common Vampire Bat (*Desmodus rotundus*)	2	6, 18	1	8

Table S4: MEME results for each alignment including number of sites identified to be under significant diversifying selection and at which site in each alignment.				
Toxin	Species	Sites identified under positive selection	Sites number	dN/dS
Kallikren-1(KLK1) *Blarina*	Short tailed shrew (*Blarina brevicauda*)	4	20, 116, 129, 183	0.619
Kallikren-1(KLK1) *Solenodon*	Hispaniolan solenodon (*Solenodon paradoxus*)	6	41, 49, 103, 152, 178, 224	0.751
Kallikrein-1 (KLK1) Eulipotyphla	Short tailed shrew (*Blarina brevicauda*)<br><br>Hispaniolan solenodon (*Solenodon paradoxus*)	16	4, 12, 21, 46, 67, 72, 78, 117, 127, 135, 183, 197, 210, 217, 256, 258,	0.593
Kallikren-1 (KLK1) All	Short tailed shrew (*Blarina brevicauda*)<br><br>Hispaniolan solenodon (*Solenodon paradoxus*)<br><br>Common Vampire Bat (*Desmodus rotundus*)	21	5, 10, 55, 58, 60, 61, 62, 65, 71, 74, 86, 109, 117, 138, 165, 179, 192, 199, 236, 238, 240	0.513
Plasminogen Activator	Hairy legged vampire bat (*Diphylla ecaudata*)<br><br>White winged vampire bat (*Diaemus youngi*)<br><br>Common vampire bat (*Desmodus rotundus*)	4	40, 184, 211, 342	1.04
Desmallipins (Desmodus allergen-related lipocalins)	Common Vampire Bat (*Desmodus rotundus*)	15	16, 30, 37, 43, 79, 87, 89, 121, 125, 130, 155, 167, 174, 178, 185	1.18

PACAP (Pituitary Adenylate Cyclase-Activating Polypeptide)	Common Vampire Bat (*Desmodus rotundus*)	2	4, 41	1.04
Cysteine-rich secretory proteins (CRiSP) *Desmodus*	Common Vampire Bat (*Desmodus rotundus*)	0	N/A	$\omega = 1.62$
Cysteine-rich secretory proteins (CRiSP) All	Common Vampire Bat (*Desmodus rotundus*)<br><br>Platypus (*Ornithorhynchus anatinus*)	0	N/A	$\omega = 0.482$
Kunitz Domain One	Common Vampire Bat (*Desmodus rotundus*)	1	13	$\omega = 0.444$
Kunitz Domain Two (Chiroptera)	Common Vampire Bat (*Desmodus rotundus*)	2	6, 26	$\omega = 2.51$

Table S5: CodeML results for the alignment of KLK1 *Blarina brevicauda*, including results of all models identified in this study.

Model	Likelihood	dN/dS	Parameters					Number of sites where omega > 1*
M0 (Null)	-2742.182956	0.57984	N/A					N/A
M1a (Neutral)	-2700.342314	0.5481	P_0: 0.482 ω_0: 0.06		P_1: 0.518 ω_1:1.0			N/A
M2a†	-2694.060310	0.8139	P_0: 0.522 ω_0: 0.11		P_1: 0.324 ω_1: 1.0		P_2: 0.154 ω_2: 2.81	None
M3†	-2693.831198	0.8055	P_0: 0.288 ω_0: 0.0		P_1: 0.484 ω_1: 0.51		P_2: 0.227 ω_2: 2.45	N/A
M7 (beta)	-2701.596702	0.5558	p: 0.10166					N/A
			q: 0.08126					
M8 (beta and ω)†	-2693.907033	0.8075	p0: 0.804	P = 0.380	q = 0.629	P_1 = 0.196	ω = 2.58	13 (P>0.95) (12, 20, 49, 84, 90, 111, 113, 117, 136, 172, 240, 244, 247)<br><br>3 (P>0.99) (75, 121, 171)
M8a†	-2700.358698	0.5481	p0: 0.482	P = 6.81	q = 99	P_1 = 0.517	ω = 1.00	N/A

† Models that allow ω > 1 (checking for diversifying selection)
*identified by the Bayes Empirical Bayes (BEB) model

Table S6: Significance of CodeML results for the alignment of KLK1 *Blarina brevicauda*

Null model vs Alternative model		X2 result	K result	Level of significance
M0	M3	96.7	4	P < 0.001 (0.000000000)
M1a	M2a	12.6	2	P < 0.001 (0.001869650)
M7	M8	15.4	2	P < 0.001 (0.000457530)
M8a	M8	12.9	1	P < 0.001 (0.000327998)

Table S7: CodeML results for the alignment of KLK1 *Solenodon paradoxus*, including results of all models identified in this study.

Model	Likelihood	dN/dS	Parameters					Number of sites where omega > 1*
M0 (Null)	-1825.959506	0.58254	N/A					N/A
M1a (Neutral)	-1794.224892	0.4647	P_0: 0.555 ω_0: 0.04	P_1: 0.445 ω_1:1.0				N/A
M2a†	-1783.399757	0.7298	P_0: 0.483 ω_0: 0.005	P_1 0.473 ω_1: 1.0	P_2: 0.044 ω_2: 5.85			1 (P>0.95) (48) 2 (P>0.99) (98, 163)
M3†	-1783.295718	0.7002	P_0: 0.462 ω_0: 0.000	P_1: 0.487 ω_1: 0.89	P_2: 0.05 ω_2: 5.25			N/A
M7 (beta)	-1794.457015	0.4416	p: 0.01396 q: 0.01724					N/A
M8 (beta and ω)†	-1783.338167	0.7073	p0: 0.954	P = 0.04	q = 0.05	P_1 = 0.05	ω = 5.55	2 (P>0.95) (119, 167) 4 (P>0.99) (48, 98, 163, 177)
M8a†	-1794.201112	0.4615	p0: 0.565	P = 0.137	q = 1.96	P_1 = 0.435	ω = 1.0	N/A

† Models that allow ω > 1 (checking for diversifying selection)
*identified by the Bayes Empirical Bayes (BEB) model

Table S6: Significance of CodeML results for the alignment of KLK1 *Solenodon paradoxus*

Null model vs Alternative model		X2 result	K result	Level of significance
M0	M3	85	4	P < 0.001 (0.000000000)
M1a	M2a	21.7	2	P < 0.001 (0.000019893)
M7	M8	22.2	2	P < 0.001 (0.000014830)
M8a	M8	21.7	1	P < 0.001 (0.000003145)

Table S9: CodeML results for the alignment of KLK1 Eulipotyphla, including results of all models identified in this study.

Model	Likelihood	dN/dS	Parameters	Number of sites where omega > 1*
M0 (Null)	-3254.134397	0.49379	N/A	N/A
M1a (Neutral)	-3181.163308	0.5066	P_0: 0.569 ω_0: 0.13; P_1: 0.430 ω_1:1.0	N/A
M2a†	-3167.738986	0.6748	P_0: 0.533 ω_0: 0.132; P_1: 0.436 ω_1: 1.0; P_2: 0.03 ω_2: 5.60	1 (P>0.95) (90) 2 (P>0.99) (111, 155)
M3†	-3167.735906	0.6713	P_0: 0. 530 ω_0: 0.13; P_1 0.440 ω_1: 0.99; P_2:0.03 ω_2: 5.53	N/A
M7 (beta)	-3181.985664	0.4700	p: 0.36708; q: 0.41393	N/A
M8 (beta and ω)†	-3167.205706	0.6260	p0: 0.963; P = 0.392; q = 0.44; P_1 = 0.04; ω = 4.69	3 (P>0.95) (87, 90, 151) 2 (P>0.99) (111, 155)
M8a†	-3179.158058	0.4761	p0: 0.641; P = 0.856; q = 3.60; P_1 = 0.36; ω = 1	N/A

† Models that allow ω > 1 (checking for diversifying selection)
*identified by the Bayes Empirical Bayes (BEB) model

Table S10: Significance of CodeML results for the alignment of KLK1 Eulipotyphla

Null model vs Alternative model		X2 result	K result	Level of significance
M0	M3	172.8	4	P < 0.001 (0.000000000)
M1a	M2a	26.9	2	P < 0.001 (0.000001479)
M7	M8	29.6	2	P < 0.001 (0.000000381)
M8a	M8	23.9	1	P < 0.001 (0.000001012)

Table S11: CodeML results for the alignment of KLK1 All, including results of all models identified in this study.

Model	Likelihood	dN/dS	Parameters					Number of sites where omega > 1*
M0 (Null)	-2982.511043	0.43905	N/A					N/A
M1a (Neutral)	-2910.059147	0.5147	P_0: 0.545 ω_0: 0.11			P_1: 0.456 ω_1:1.0		N/A
M2a†	-2904.636186	0.6339	P_0: 0.523 ω_0: 0.11		P_1: 0.436 ω_1: 1.0		P_2: 0.04 ω_2: 3.44	1 (P>0.95) (88)
M3†	-2901.128891	0.5587	P0: 0.222 ω_0:0.0	P0: 0.329 ω_0:0.22	P_1: 0.397 ω_1:0.85		P_2: 0.052 ω_2:2.86	N/A
M7 (beta)	-2908.090763	0.4537	p: 0.32733<br>q: 0.39411					N/A
M8 (beta and ω)†	-2901.550405	0.5592	p0: 0.949	P = 0.364	q = 0.474	P_1 = 0.05	ω = 2.87	3 (P>0.95) (103, 108, 148) 1 (P>0.99) (88)
M8a†	-2906.989456	0.4649	p0 = 0.664	P = 0.549	q = 2.13	P_1 = 0.336	ω = 1.00	N/A

† Models that allow ω > 1 (checking for diversifying selection)
*identified by the Bayes Empirical Bayes (BEB) model

Table S12: Significance of CodeML results for the alignment of KLK1 All

Null model vs Alternative model		X2 result	K result	Level of significance
M0	M3	162.8	6	P < 0.001 (0.000000000)
M1a	M2a	10.8	2	P < 0.01 (0.004414057)
M7	M8	13.1	2	P < 0.001 (0.001443971)
M8a	M8	10.9	1	P < 0.001 (0.000973079)

<table>
<tr><td colspan="9">Table S13: CodeML results for the alignment of Plasminogen Activator, including results of all models identified in this study.</td></tr>
<tr><th>Model</th><th>Likelihood</th><th>dN/dS</th><th colspan="5">Parameters</th><th>Number of sites where omega > 1*</th></tr>
<tr><td>M0 (Null)</td><td>-2402.641004</td><td>1.0549</td><td colspan="5">N/A</td><td>N/A</td></tr>
<tr><td>M1a (Neutral)</td><td>-2376.314343</td><td>0.4849</td><td colspan="2">P_0: 0.515
ω_0: 0.0</td><td colspan="3">P_1: 0.485
ω_1:1.0</td><td>N/A</td></tr>
<tr><td>M2a†</td><td>-2351.136942</td><td>1.3046</td><td colspan="2">P_0: 0. 708
ω_0: 0.163</td><td colspan="2">P_1: 0. 05
ω_1: 1.0</td><td>P_2: 0.24
ω_2: 4.74</td><td>10 (P>0.95)
(13, 24, 30, 133, 169, 196, 219,244, 283, 303)
4 (P>0.99)
(12, 39,107, 243)</td></tr>
<tr><td>M3†</td><td>-2350.557836</td><td>1.3338</td><td colspan="2">P_0: 0. 711
ω_0: 0.139</td><td colspan="2">P_1: 0. 270
ω_1: 3.73</td><td>P_2: 0.02
ω_2: 12.3</td><td>N/A</td></tr>
<tr><td>M7 (beta)</td><td>-2376.357489</td><td>0.5000</td><td colspan="5">p: 0.00500
q: 0.00500</td><td>N/A</td></tr>
<tr><td>M8 (beta and ω)†</td><td>-2351.139833</td><td>1.3082</td><td>p0: 0.752</td><td>P = 23.8</td><td>q = 99</td><td>P_1 = 0.248</td><td>ω = 4.68</td><td>14 (P>0.95)
(24, 28, 30, 76, 77, 135, 137, 156, 169, 195, 219, 280, 298, 302)
10 (P>0.99)
(12, 13, 39, 107, 133, 218, 243, 244, 283, 303)</td></tr>
<tr><td>M8a†</td><td>-2376.363134</td><td>0.4849</td><td>p0: 0.515</td><td>P = 0.005</td><td>q = 1.72</td><td>P_1 = 0.49</td><td>ω = 1.00</td><td>N/A</td></tr>
<tr><td colspan="9">† Models that allow ω > 1 (checking for diversifying selection)
*identified by the Bayes Empirical Bayes (BEB) model</td></tr>
</table>

Table S14: Significance of CodeML results for the alignment of Plasminogen Activator

Null model vs Alternative model		X2 result	K result	Level of significance
M0	M3	104.2	4	P < 0.001 (0.000000000)
M1a	M2a	50.4	2	P < 0.001 (0.000000000)
M7	M8	50.4	2	P < 0.001 (0.000000000)
M8a	M8	125.6	15	P < 0.001 (0.000000000)

Table S15: CodeML results for the alignment of Desmallipins, including results of all models identified in this study.

<table>
<tr><th>Model</th><th>Likelihood</th><th>dN/dS</th><th colspan="5">Parameters</th><th>Number of sites where omega > 1*</th></tr>
<tr><td>M0 (Null)</td><td>-2492.789614</td><td>1.09464</td><td colspan="5">N/A</td><td>N/A</td></tr>
<tr><td>M1a (Neutral)</td><td>-2454.891429</td><td>0.6265</td><td colspan="3">P_0: 0.418
ω_0: 0.11</td><td colspan="2">P_1: 0.582
ω_1:1.0</td><td>N/A</td></tr>
<tr><td>M2a†</td><td>-2432.882104</td><td>1.3216</td><td colspan="2">P_0: 0. 347
ω_0: 0.153</td><td colspan="2">P_1: 0.398
ω_1: 1.0</td><td>P_2:0.255
ω_2: 3.47</td><td>3 (P>0.95)
(15, 42, 74)
7 (P>0.99)
(1, 28, 64, 110, 146, 159, 160)</td></tr>
<tr><td>M3†</td><td>-2432.627521</td><td>1.3623</td><td colspan="2">P_0: 0. 503
ω_0: 0.27</td><td colspan="2">P_1 0.370
ω_1: 1.78</td><td>P_2:0.126
ω_2: 4.51</td><td>N/A</td></tr>
<tr><td rowspan="2">M7 (beta)</td><td rowspan="2">-2458.205228</td><td rowspan="2">0.6304</td><td colspan="5">p: 0.01630</td><td rowspan="2">N/A</td></tr>
<tr><td colspan="5">q: 0.00896</td></tr>
<tr><td>M8 (beta and ω)†</td><td>-2432.885088</td><td>1.3393</td><td>p0: 0.725</td><td>P = 0.420</td><td>q = 0.321</td><td>P_1 = 0.28</td><td>ω = 3.37</td><td>17 (P>0.95)
(2, 12, 24, 25, 27, 46, 47, 69, 78, 106, 121, 140, 152, 155, 157, 161, 164)
12 (P>0.99)
(1, 7, 15, 28, 42, 61, 64, 74, 110, 146, 159, 160)</td></tr>
<tr><td>M8a†</td><td>-2454.916520</td><td>0.6265</td><td>p0: 0.418</td><td>P = 11.99</td><td>q = 99</td><td>P_1 = 0.581</td><td>ω = 1</td><td></td></tr>
</table>

† Models that allow ω > 1 (checking for diversifying selection)
*identified by the Bayes Empirical Bayes (BEB) model

Table S16: Significance of CodeML results for the alignment of Desmallipins

Null model vs Alternative model		X2 result	K result	Level of significance
M0	M3	120.3	4	P < 0.001 (0.000000000)
M1a	M2a	44	2	P < 0.001 (0.000000000)
M7	M8	50.6	2	P < 0.001 (0.000000000)
M8a	M8	44.1	1	P < 0.001 (0.000000000)

<table>
<tr><td colspan="9">Table S17: CodeML results for the alignment of PACAP, including results of all models identified in this study.</td></tr>
<tr><th>Model</th><th>Likelihood</th><th>dN/dS</th><th colspan="5">Parameters</th><th>Number of sites where omega > 1*</th></tr>
<tr><td>M0 (Null)</td><td>-1300.669556</td><td>0.90099</td><td colspan="5">N/A</td><td>N/A</td></tr>
<tr><td>M1a (Neutral)</td><td>-1289.424644</td><td>0.5148</td><td colspan="2">P_0: 0.485
ω0: 0.0</td><td colspan="3">P_1: 0.515
$ω_1$:1.0</td><td>N/A</td></tr>
<tr><td>M2a†</td><td>-1280.254233</td><td>1.3239</td><td colspan="2">P_0: 0. 459
$ω_0$: 0.0</td><td colspan="2">P_1: 0.389
$ω_1$: 1.0</td><td>P_2:0.151
$ω_2$: 6.16</td><td>4 (P>0.95)
(4, 164, 165, 170)
1 (P>0.99)
(20)</td></tr>
<tr><td>M3†</td><td>-1280.017379</td><td>1.3933</td><td colspan="2">P_0: 0. 545
$ω_0$: 0.0</td><td colspan="2">P_1: 0.376
$ω_1$: 1.83</td><td>P_2:0.078
$ω_2$: 8.82</td><td>N/A</td></tr>
<tr><td rowspan="2">M7 (beta)</td><td rowspan="2">-1289.446181</td><td rowspan="2">0.5000</td><td colspan="5">p: 0.00503</td><td rowspan="2">N/A</td></tr>
<tr><td colspan="5">q: 0.00504</td></tr>
<tr><td>M8 (beta and ω)†</td><td>-1280.298166</td><td>1.3208</td><td>p0: 0.840</td><td>P = 0.013</td><td>q = 0.016</td><td>P_1 = 0.160</td><td>ω = 5.96</td><td>14 (P>0.95)
(1, 7, 12, 13, 39, 40, 43, 66, 68, 87, 91, 100, 105, 107)</td></tr>
<tr><td>M8a†</td><td>-1289.424628</td><td>0.5148</td><td>p0: 0.485</td><td>P = 0.005</td><td>q = 1.83</td><td>P_1 = 0.514</td><td>ω = 1.00</td><td>N/A</td></tr>
<tr><td colspan="9">† Models that allow ω > 1 (checking for diversifying selection)
*identified by the Bayes Empirical Bayes (BEB) model</td></tr>
</table>

Table S18: Significance of CodeML results for the alignment of PACAP

Null model vs Alternative model		X2 result	K result	Level of significance
M0	M3	41.3	4	P < 0.001 (0.000000023)
M1a	M2a	18.3	2	P < 0.001 (0.000104074)
M7	M8	18.3	2	P < 0.001 (0.000106431)
M8a	M8	18.3	1	P < 0.001 (0.000019343)

Table S19: CodeML results for the alignment of CRiSP *Desmodus*, including results of all models identified in this study.

Model	Likelihood	dN/dS	Parameters					Number of sites where omega > 1*
M0 (Null)	-671.081776	1.48836	N/A					N/A
M1a(Neutral)	-668.553921	0.4589	P_0: 0.541 ω_0: 0.0	P_1: 0.459 ω_1:1.0				N/A
M2a†	-660.883331	2.0592	P_0: 0. 905 ω_0: 0.58	P_1: 0.0 ω_1: 1.0	P_2:0.1 ω_2: 16.125			4 (P>0.95) (7, 13, 39, 66)
M3†	-660.883331	1.4884	P_0: 0.760 ω_0: 0.58	P_1: 0.145 ω_1: 0.58	P_2:0.1 ω_2: 16.125			N/A
M7 (beta)	-668.591488	0.5000	p: 0.00500 q: 0.00501					N/A
M8 (beta and ω)†	-660.884052	2.0806	p0: 0.906	P = 99	q = 69.09	P_1= 0.094	ω = 16.36	4 (P>0.95) (4, 164, 165, 169) 2 (P>0.99) (20, 170)
M8a†	-668.553916	0.4589	p0: 0.541	P = 0.01	q = 1.1	P_1 = 0.459	ω = 1	N/A

† Models that allow ω > 1 (checking for diversifying selection)
*identified by the Bayes Empirical Bayes (BEB) model

Table S20: Significance of CodeML results for the alignment of CRiSP *Desmodus*

Null model vs Alternative model		X2 result	K result	Level of significance
M0	M3	20.4	4	P < 0.001 (0.000416898)
M1a	M2a	15.3	2	P < 0.001 (0.000466343)
M7	M8	15.4	2	P < 0.001 (0.000449472)
M8a	M8	15.3	1	P < 0.001 (0.000089808)

Table S21: CodeML results for the alignment of CRiSP All, including results of all models identified in this study

Model	Likelihood	dN/dS	Parameters	Number of sites where omega > 1*
M0 (Null)	-1031.729998	0.34629	N/A	N/A
M1a (Neutral)	-1008.617620	0.4574	P_0: 0.568 ω_0: 0.04<br>P_1: 0.432 ω_1:1.0	N/A
M2a†	-1002.587333	1.3843	P_0: 0. 574 ω_0: 0.05<br>P_1: 0.34 ω_1: 1.0<br>P_2:0.09 ω_2: 11.52	3(P>0.95)<br>(7, 13, 64)
M3†	-1002.587333	1.4772	P_0: 0. 598 ω_0: 0.06<br>P_1: 0.316 ω_1: 1.19<br>P_2:0.09 ω_2: 12.43	N/A
M7 (beta)	-1009.248073	0.4542	p: 0.11168<br>q: 0.13422	N/A
M8 (beta and ω)†	-1002.784971	1.2923	p0: 0.91<br>P = 0.171<br>q = 0.3<br>P_1 = 0.09<br>ω = 10.69	4 (P>0.95)<br>(39, 66, 89, 98)<br>3 (P>0.99)<br>(7, 13, 64)
M8a†	-1008.623987	0.4567	p0: 0.569<br>P = 4.78<br>q = 99<br>P_1 = 0.43<br>ω = 1.00	

† Models that allow ω > 1 (checking for diversifying selection)
*identified by the Bayes Empirical Bayes (BEB) model

Table S22: Significance of CodeML results for the alignment of CRiSP All

Null model vs Alternative model		X2 result	K result	Level of significance
M0	M3	58.3	4	P < 0.001 (0.000000000)
M1a	M2a	12.1	3	P < 0.01 (0.007178514)
M7	M8	12.9	2	P < 0.01 (0.001559949)
M8a	M8	11.7	1	P < 0.001 (0.000632424)

Table S23: CodeML results for the alignment of Kunitz Domain One, including results of all models identified in this study

Model	Likelihood	dN/dS	Parameters					Number of sites where omega > 1*
M0 (Null)	-686.243223	0.29323	N/A					N/A
M1a (Neutral)	-650.130441	0.5524	P_0: 0.455<br>ω_0: 0.02	P_1: 0.545<br>ω_1:1.0				N/A
M2a†	-647.141997	1.4170	P_0: 0. 444<br>ω_0: 0.015	P_1: 0.403<br>ω_1: 1.0	P_2:0.15<br>ω_2: 6.56			1(P>0.99)<br>28
M3†	-645.421643	1.4033	P_0: 0. 384<br>ω_0: 0.0	P_1: 0.304<br>ω_1: 0.33	P_2:0.312<br>ω_2: 4.18			
M7 (beta)	-649.398512	0.4602	p: 0.13042<br>q: 0.13042					N/A
M8 (beta and ω)†	-646.307806	1.3985	p0: 0.07	P = 0.18	q = 0.6	P_1 = 0.28	ω = 4.49	2 (P>0.95)<br>(42, 43)<br>2 (P>0.99)<br>(28, 38)
M8a†	-649.149486	0.4661	p0: 0.585	P = 0.148	q = 1.24	P_1 = 0.42	ω = 1.00	N/A

† Models that allow ω > 1 (checking for diversifying selection)
*identified by the Bayes Empirical Bayes (BEB) model

Table S24: Significance of CodeML results for the alignment of Kunitz Domain One

Null model vs Alternative model		X2 result	K result	Level of significance
M0	M3	81.6	4	P < 0.001 (0.000000000)
M1a	M2a	6	2	P < 0.05 (0.050365745)
M7	M8	6.2	2	P < 0.05 (0.045469841)
M8a	M8	5.9	1	P < 0.05 (0.017126540)

Table S25: CodeML results for the alignment of Kunitz Domain Two, including results of all models identified in this study

Model	Likelihood	dN/dS	Parameters					Number of sites where omega > 1*
M0 (Null)	-490.153926	2.28040	N/A					N/A
M1a (Neutral)	-490.985448	0.7345	P_0: 0.266 ω_0: 0.00		P_1: 0.735 ω_1:1.0			N/A
M2a†	-484.686490	2.6283	P_0: 0.468 ω_0: 0.0		P_1: 0.0 ω_1: 1.0		P_2:0.532 ω_2: 4.94	3 (P>0.95) (15, 18, 52) 1 (P>0.99) (6)
M3†	-484.686490	2.6283	P_0: 0.468 ω_0: 0.0		P_1: 0.366 ω_1: 4.94		P_2:0.166 ω_2: 4.94	N/A
M7 (beta)	-491.075889	0.8000	p: 0.02008					N/A
			q: 0.00500					
M8 (beta and ω)†	-484.686489	2.6283	p0: 0.468	P = 0.005	q = 12.14	P_1 = 0.53	ω = 4.938	4 (P>0.95) (31, 32, 50, 52) 3 (P>0.99) (6, 15, 18)
M8a†	-490.985439	0.7345	p0: 0.266	P = 0.005	q = 1.58	P_1 = 0.735	ω = 1.00	N/A

† Models that allow $\omega > 1$ (checking for diversifying selection)
*identified by the Bayes Empirical Bayes (BEB) model

Table S26: Significance of CodeML results for the alignment of Kunitz Domain Two

Null model vs Alternative model		X2 result	K result	Level of significance
M0	M3	10.9	4	$P < 0.05$ (0.027305797)
M1a	M2a	12.6	2	$P < 0.01$ (0.001838219)
M7	M8	12.6	2	$P < 0.01$ (0.001838217)
M8a	M8	12.6	1	$P < 0.001$ (0.000386180)

Table S27: TreeSAAP values for each alignment, including magnitude, the value and the site number corresponding to each alignment. Sites with some level of property but at a non-significant value were included as well for completeness.						
Toxin	Species	TreeSAAP property	Magnitude	Value	Site number and value	Non-significant sites identified
Kallikren-1(KLK1) *Blarina*	Short tailed shrew (*Blarina brevicauda*)	Isoelectric point	8	4.767	181(3.211)	N/A
		Power to be at the C-terminal	7	3.849	88 (3.672), 90(3.398), 118 (3.254) and 200 (4.319)	N/A
Kallikren-1(KLK1) *Solenodon*	Hispaniolan solenodon (*Solenodon paradoxus*)	Power to be at the middle of alpha-helix	7	3.598	154 (3.354)	N/A
Kallikrein-1 (KLK1) Eulipotyphla	Short tailed shrew (*Blarina brevicauda*) Hispaniolan solenodon (*Solenodon paradoxus*)	Hydropathy	6	3.212	129(4.122), 185 (4.69) and 208 (3.583)	N/A
		Power to be at the C-terminal	6	5.718	85 (3.101), 114(3.671), 127(3.581), 178(3.128) and 202 (4.449)	10 (magnitude 8), 208 (magnitude 7) and 209 (magnitude 7)
Kallikren-1 (KLK1) All	Short tailed shrew (*Blarina brevicauda*) Hispaniolan solenodon (*Solenodon paradoxus*) Common Vampire Bat (*Desmodus rotundus*)	Hydropathy	6	3.182	165(3.915) and 190 (3.7)	171 (magnitude 8)
		Power to be at the C-terminal	6	6.497	78 (3.601), 107 (3.1), 109 (3.718), and 184 (4.202)	190 (magnitude 7)
		Power to be at the middle of alpha-helix	7	3.552	N/A	N/A
Plasminogen Activator	Hairy legged vampire bat (*Diphylla ecaudata*) White winged vampire bat (*Diaemus youngi*)	Isoeletric point	8	3.672	N/A	N/A

	Common vampire bat (*Desmodus rotundus*)					
Desmallipins (Desmodus allergen-related lipocalins)	Common Vampire Bat (*Desmodus rotundus*)	Isoeletric point	8	4.826	106 (5.322)	42 (Magnitude 7)
PACAP (Pituitary Adenylate Cyclase-Activating Polypeptide)	Common Vampire Bat (*Desmodus rotundus*)	N/A				
Cysteine-rich secretory proteins (CRiSP) *Desmodus*	Common Vampire Bat (*Desmodus rotundus*)	Long-range non-bonded energy	8	3.398	7(3.211)	N/A
		Polar requirement	7	3.939	N/A	N/A
Cysteine-rich secretory proteins (CRiSP) All	Common Vampire Bat (*Desmodus rotundus*) Platypus (*Ornithorhynchus anatinus*)	Isoeletric point	8	3.8	28 (3.237)	N/A
		Polar requirement	7	4.326	N/A	N/A
		Power to be at the C-terminal	6	5.064	N/A	N/A
Kunitz Domain One	Common Vampire Bat (*Desmodus rotundus*)	Isoelectric point	7	4.711	N/A	N/A
		Partial specific volume	6	3.74	N/A	N/A
Kunitz Domain Two	Common Vampire Bat (*Desmodus rotundus*)	Normalized consensus hydrophobicity	8	3.253	N/A	N/A
		Power to be at the N-terminal	6	3.64	N/A	N/A

Table S28: Model selection result from MEGA for maximum likelihood tree for each alignment		
Toxin	Species	Model
Kallikren-1(KLK1) *Blarina*	Short tailed shrew (*Blarina brevicauda*)	T92+I
Kallikren-1(KLK1) *Solenodon*	Hispaniolan solenodon (*Solenodon paradoxus*)	T92+G
Kallikrein-1 (KLK1) Eulipotyphla	Short tailed shrew (*Blarina brevicauda*)<br>Hispaniolan solenodon (*Solenodon paradoxus*)	T92+G
Kallikren-1 (KLK1) All	Short tailed shrew (*Blarina brevicauda*)<br>Hispaniolan solenodon (*Solenodon paradoxus*)<br>Common Vampire Bat (*Desmodus rotundus*)	T92+G
Plasminogen Activator	Hairy legged vampire bat (*Diphylla ecaudata*)<br>White winged vampire bat (*Diaemus youngi*)<br>Common vampire bat (*Desmodus rotundus*)	K2+G
Desmallipins (*Desmodus* allergen-related lipocalins)	Common Vampire Bat (*Desmodus rotundus*)	K2+G
PACAP (Pituitary Adenylate Cyclase-Activating Polypeptide)	Common Vampire Bat (*Desmodus rotundus*)	HKY+G
Cysteine-rich secretory proteins (CRiSP) *Desmodus*	Common Vampire Bat (*Desmodus rotundus*)	T92+G
Cysteine-rich secretory proteins (CRiSP) *Desmodus* and *Ornithorhynchus*	Common Vampire Bat (*Desmodus rotundus*)<br>Platypus (*Ornithorhynchus anatinus*)	T92
Kunitz Domain One	Common Vampire Bat (*Desmodus rotundus*)	JC + I
Kunitz Domain Two	Common Vampire Bat (*Desmodus rotundus*)	JC +G
